# Target-Guided Isolation of *O*-tigloylcyclovirobuxeine-B from *Buxus* *sempervirens* L. by Centrifugal Partition Chromatography

**DOI:** 10.3390/molecules25204804

**Published:** 2020-10-19

**Authors:** Lara U. Szabó, Thomas J. Schmidt

**Affiliations:** Institute of Pharmaceutical Biology and Phytochemistry (IPBP), University of Münster, Pharma Campus Correnstraße 48, D-48149 Münster, Germany; lszabo@uni-muenster.de

**Keywords:** *Buxussempervirens* L., *nor*-cycloartane alkaloids, *O*-tigloylcyclovirobuxeine-B, antimalarial activity, target-guided isolation, centrifugal partition chromatography

## Abstract

The increasing drug resistance of malaria parasites challenges the treatment of this life-threatening disease. Consequently, the development of innovative and effective antimalarial drugs is inevitable. *O*-tigloylcyclovirobuxeine-B, a *nor*-cycloartane alkaloid from *Buxus* *sempervirens* L., has shown promising and selective in vitro activity in previous studies against *Plasmodium* *falciparum* (*Pf*), causative agent of Malaria tropica. For further investigations, it is indispensable to develop an advanced and efficient isolation procedure of this valuable natural product. Accordingly, we used liquid–liquid chromatography including centrifugal partition chromatography (CPC) to obtain the pure alkaloid on a semi-preparative scale. Identification and characterization of the target compound was accomplished by UHPLC/+ESI-QqTOF-MS/MS, ^1^H NMR and ^13^C NMR. In conclusion, this work provides a new and efficient method to obtain *O*-tigloylcyclovirobuxeine-B, a valuable natural product, as a promising antiplasmodial lead structure for the development of innovative and safe medicinal agents.

## 1. Introduction

*Buxus sempervirens* L. (European Box; Buxaceae) is an indeciduous shrub, which represents a rich source for *nor*-triterpene alkaloids of the *nor*-cycloartane type. Decoctions of the leaves are well known in folk medicine for a variety of indications, including malaria [1,2]. Malaria is a poverty-related infectious disease caused by protozoans of the genus *Plasmodium* and transmitted by infected female *Anopheles* mosquitoes. Worldwide there were 228 million estimated malaria cases in 2018 [3]. The development of new effective agents against this life-threatening disease is imperative because of increasing drug resistance.

Extracts of the leaves from *B. sempervirens* L. have shown selective in vitro activity against *Plasmodium falciparum* that causes the majority of malaria deaths [4,5,6]. In 2014, Althaus et al. obtained a small amount of *O*-tigloylcyclovirobuxeine-B from an alkaloid-enriched fraction of *B. sempervirens* leaf extract by bioactivity-guided isolation. The natural product yielded an IC_50_ value against *Pf* of 0.46 µg/mL (0.92 µM) vs. 9.4 µg/mL (18.9 µM) for cytotoxicity against L6 rat cells [5]. Consequently, *O*-tigloylcyclovirobuxeine-B is a promising lead compound for the development of novel and safe medicinal agents against malaria. For further investigations, including in vivo studies, it is required to establish a simple, reproducible and productive isolation procedure for this valuable compound. For this purpose, we describe a new efficient semi-preparative scale isolation of *O*-tigloylcyclovirobuxeine-B using only liquid–liquid partition chromatography. The authenticity of the isolated *O*-tigloylcyclovirobuxeine-B was proven by ^1^H NMR, ^13^C NMR and +ESI-QqTOF-MS/MS.

## 2. Results and Discussion

We established an advanced and improved isolation method of *O*-tigloylcyclovirobuxeine-B (**1**) without using a solid stationary phase. After extraction of about 1.5 kg of dried plant material, only three steps of liquid–liquid partition (one acid/base partitioning step and two steps of centrifugal partition chromatography (CPC)) were required in order to obtain 35 mg of target compound.

The advantages in using this all-liquid separation scheme are diverse, including a high recovery of injected sample, a low solvent consumption (less waste and expense), minimized tailing of eluted peaks, no irreversible loss of sample due to chemisorption, high reproducibility and high purification levels [7,8,9].

### 2.1. Target-Guided Isolation of O-tigloylcyclovirobuxeine-B (1)

#### 2.1.1. Extraction and Alkaloid-Enrichment of *B. sempervirens* L. Leaves

**Figure 1:**
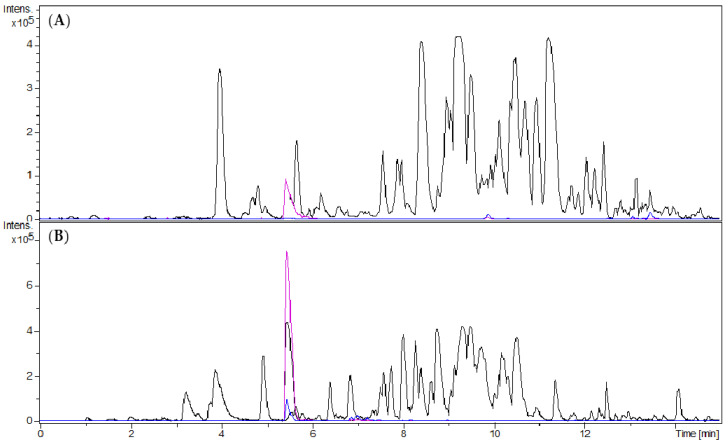
UHPLC/+ESI-QqTOF-MS chromatograms: Base peak chromatogram of *m/z* 200–1000 (black), Extracted ion chromatogram of *m/z* 249 [M + 2H]^2+^ (pink), Extracted ion chromatogram of *m/z* 497 [M + H]^+^ (blue); (**A**) dichloromethane extract (GBUS); (**B**) alkaloid fraction (ALOF). The target compound was enriched from GBUS to ALOF by factor 19.

The discovery of the target compound as antiplasmodial principle of B. sempervirens leaves followed the observation of an increased antiplasmodial activity of an alkaloid-enriched fraction in comparison to the crude dichloromethane (DCM) extract [5]. The alkaloid-enriched fraction was obtained by acid/base extraction of the crude DCM extract. In the present study, we obtained 4.63 g of the alkaloid fraction (ALOF) from 112 g of crude DCM extract (GBUS) from 1.47 kg of plant material. Compared to the crude extract, a 19-fold enrichment of O-tigloylcyclovirobuxeine-B (1) was observed in UHPLC/+ESI-QqTOF-MS (henceforth termed “LC/MS”) analysis (Figure 1).

#### 2.1.2. Selection of Suitable Two-Phase Solvent Systems

In order to separate a sample successfully by CPC the biphasic solvent system should satisfy some crucial requirements: For an adequate retention of the stationary phase, the settling time should be shorter than 30 s [7]. The distribution coefficient (termed K_C_ according to [10]) of the target compound(s) between the two phases should be close to one (0.5 ≤ K ≤ 1.0) [8]. A smaller K_C_ value tends to give a less efficient peak resolution while a larger K_C_ value may result in broader peaks due to protracted elution time. Hence the target compound(s) should show a close to equal distribution between the upper and lower phases [8,9].

To choose an appropriate biphasic solvent system for the separation, the required distribution of the alkaloids was evaluated by thin layer chromatography (TLC) and LC/MS analysis (ALOF and fraction 2). In addition, the phase systems were assessed with regard to a stable and rapid (<30 s) phase separation.

We observed that the eluent system Hexane:EtOAc/MeOH:H_2_O (7:3/7:3) (*v*/*v*/*v*/*v*) can provide a partition appropriate for the separation of the alkaloid fraction (K_C_ value 1.16, determined by LC/MS). This two-phase system belongs to the HEMWat family (n-Hexane-Ethyl acetate-Methanol-Water) [7,11] and is suitable to separate compounds over a wide range of polarity by varying proportions of the four components.

For the subfractionation of fraction 2, the solvent system Hexane/CH_3_CN:CH_2_Cl_2_ (10/7:3) (*v*/*v*/*v*) was used and, with a K_C_ value of 0.63, ensured a partition coefficient well suitable for the target compound.

#### 2.1.3. Separation of the Alkaloid-Enriched Fraction by Centrifugal Partition Chromatography

**Figure 2:**
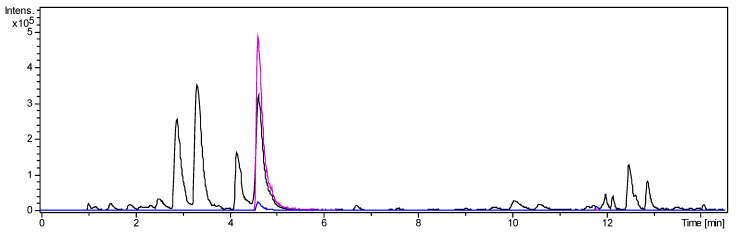
UHPLC/+ESI-QqTOF-MS chromatogram of fraction 2: Base peak chromatogram of *m/z* 200–1000 (black), Extracted ion chromatogram of *m/z* 249 [M + 2H]^2+^ (pink), Extracted ion chromatogram of *m/z* 497 [M + H]^+^ (blue). Enrichment of the target compound from ALOF to fraction 2 was 10-fold.

The alkaloid fraction was separated into 20 fractions by centrifugal partition chromatography (CPC). During elution mode, fractions 1–15 were collected and, subsequently, the extrusion phase yielded fractions 16–20. All fractions were analysed by LC/MS. Fraction 2 contained *O*-tigloylcyclovirobuxeine-B (**1**) as a main constituent (Figure 2). The major part of the target compound, enriched by factor 10 over the ALOF, was concentrated in this fraction while lower amounts were present also in fraction 1.

#### 2.1.4. Separation of Fraction 2 by Centrifugal Partition Chromatography

**Figure 3:**
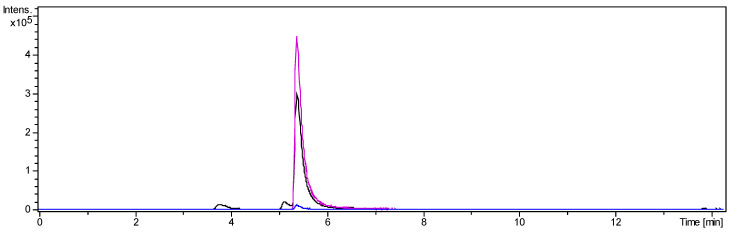
UHPLC/+ESI-QqTOF-MS chromatogram of *O*-tigloylcyclovirobuxeine-B (**1**): Base peak chromatogram of *m/z* 200–1000 (black), Extracted ion chromatogram of *m/z* 249 [M + 2H]^2+^ (pink), Extracted ion chromatogram of *m/z* 497 [M + H]^+^ (blue). Enrichment of **1** from fraction 2 was 7.8-fold.

A second CPC separation was performed to purify *O*-tigloylcyclovirobuxeine-B (**1**) from fraction 2. The target compound appeared almost quantitatively in test tubes 16 and 17. Following crystallization from acetonitrile, 35 mg fine white needles of *O*-tigloylcyclovirobuxeine-B (**1**) were obtained. The LC/MS analysis showed that the target compound was >90% pure, accompanied only by two small peaks of congeneric alkaloids (see Figure 3 and Materials and Methods, Section 3.3.3).

### 2.2. Identification of O-Tigloylcyclovirobuxeine-B (1)

The identity of the isolated compound with *O*-tigloylcyclovirobuxeine-B (**1**) (Figure 4) was confirmed by comparison of the ^1^H NMR, ^13^C NMR and +ESI-QqTOF-MS/MS spectra (Figures S2–S8, Supplementary Materials) which were in full agreement with reports in literature [5].

### 2.3. +ESI-QqTOF MS/MS fragmentation of O-tigloylcyclovirobuxeine-B (1)

**Figure 5:**
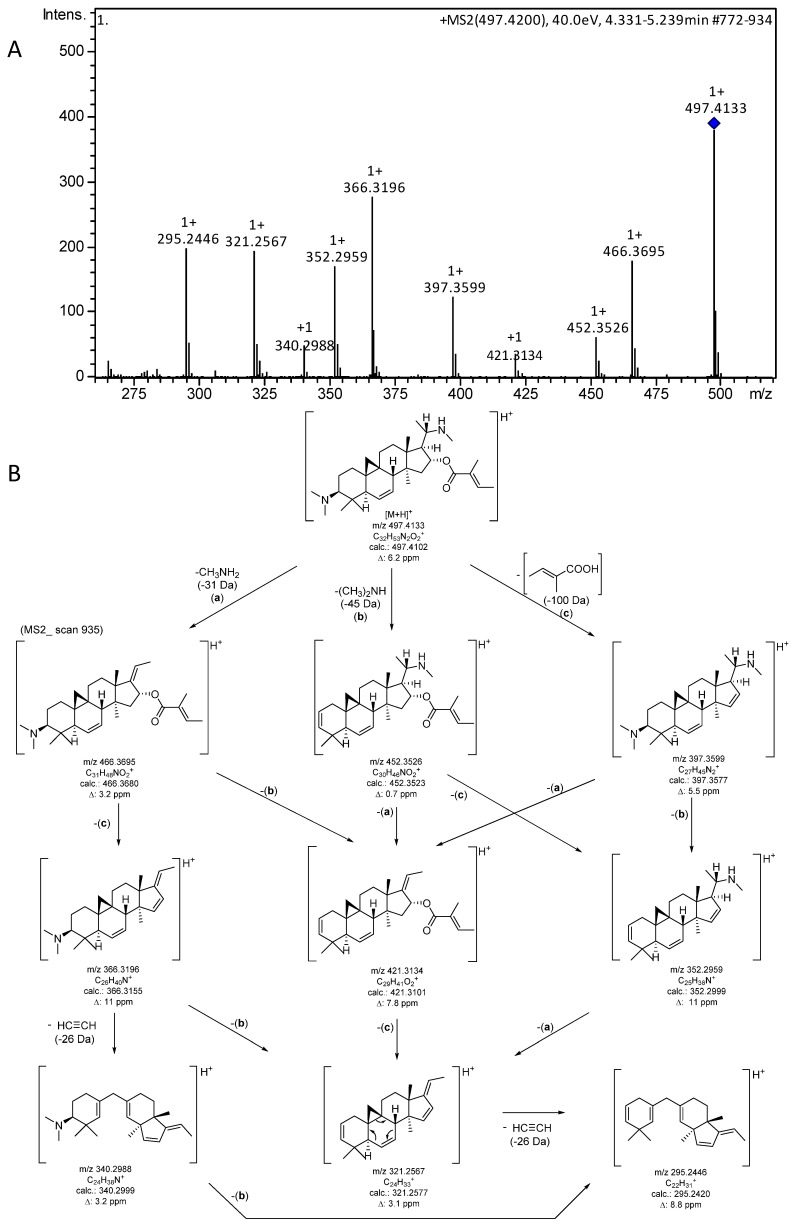
(**A**) +ESI MS/MS spectrum (CID 40 eV; only the region for Figure 5B is shown; the full spectrum is presented in Figure S8, Supplementary Materials). (**B**) Fragments and possible fragmentation pathway of the [M + H]^+^ ion of O-tigloylcyclovirobuxeine-B (**1**).

In addition to the analytical data reported previously [5], it was of interest to characterize the MS fragmentation of the target compound in order to allow unambiguous dereplication in future work (e.g., when investigating other *Buxus* species for related compounds). Therefore, MS/MS fragmentation of the singly protonated molecular ion [M + H]^+^ (*m/z* 497) was studied. The resulting mass spectrum (Figure 5A) clearly showed a very characteristic fragmentation pattern comprising all ions expected for the loss of the two amino substituents as well as the tiglic acid moiety. Furthermore, a neutral loss of 26 Da from some fragments, corresponding to C_2_H_2_ (=ethyne), appears to result from a retro-Diels-Alder-related decyclization with bond cleavage between C-5 and C-6 as well as C-7 and C-8. The fragmentation pathway along with the theoretical *m/z* values and mass deviations of the experimental signals is shown in Figure 5B.

## 3. Materials and Methods

### 3.1. Plant Material

The leaves (along with a low amount of small twigs) of *B. sempervirens* L. were collected in February 2019 on a private property in Havixbeck, Germany, from the same plants as in the previous study [5]. The identity of the plant was confirmed by T. J. Schmidt and a voucher specimen of the collection was deposited at the herbarium of the Institute of Pharmaceutical Biology and Phytochemistry, University of Münster (voucher No.: LS_BS_01 and LS_BS_02). The air-dried leaves were separated from the branches and ground with an IKA MF basic mill (IKA, Staufen, Germany) to 1 mm mesh size.

### 3.2. Extraction and Preparation of the Alklaloid-Enriched Fraction

The powdered plant material (1467 g) was extracted exhaustively in a Soxhlet-apparatus in portions of approximately 240 g with 1.5 L dichloromethane (DCM) for 36 h until the supernatant was colorless. After rotary evaporation under reduced pressure at 40 °C, a total of 112 g of crude extract (GBUS) was obtained.

An acid–base extraction was carried out to enrich the alkaloids from 103 g of the crude extract. For each extraction batch, 5 g of the extract was dissolved in 60 mL of DCM and extracted six times with 25 mL of diluted sulfuric acid R (1M, European Pharmacopoeia Reagent) in a separating funnel. Due to their increased hydrophilicity, the protonated alkaloids concentrate in the aqueous phase, while lipophilic and neutral compounds mainly accumulate in the lipophilic DCM phase. After evaporation, the DCM phase yielded 103.98 g of the lipophilic and neutral fraction (LNB). 

The collected acidic water phase was alkalized to a pH value of ≈ 10 with solid sodium hydroxide and subsequently extracted six times with 200 mL of DCM. The deprotonated alkaloids accumulate in the lipophilic phase, while hydrophilic impurities remain in the aqueous phase. Of the alkaloid fraction (ALOF), 4.63 g was obtained from the evaporated DCM phase. According to LC/MS quantification, GBUS and ALOF contained 0.06% (63.43 mg) and 1.14% (52.89 mg) of the target compound **1**, respectively, corresponding to an enrichment factor of 19. The recovery rate was 90.6% (note that only 103 of the 112 g GBUS were used).

### 3.3. Isolation of O-Tigloylcyclovirobuxeine-B (1)

#### 3.3.1. Selection of a Two-Phase Solvent System

Preliminary small-scale tests were carried out to determine a suitable phase system for the fractionation. For this purpose, 3 mg of ALOF was each mixed with 2 mL of each, the upper and lower phase, in a snap-cap glass and shaken out. The distribution of the alkaloids (see Table S1, Supplementary Materials) was visualized with Liebermann-Burchard (acetic anhydride (5 mL):sulfuric acid (5 mL):ethanol (50 mL), European Pharmacopoeia Reagent) and Anisaldehyde spray reagents (4-Methoxybenzaldehyde (0.5 mL):acetic acid (10 mL):methanol (85 mL):sulfuric acid (5 mL), European Pharmacopoeia Reagent) on a TLC plate in case of ALOF (TLC plate silica gel 60 F_254_, Merck KGaA, Darmstadt, Germany; mobile phase: butan-1-ol:H_2_O:CH_3_COOH (10:3:1) (*v*/*v*/*v*) [12]; see Figure S1, Supplementary Materials) and evaluated quantitatively by LC/MS analysis. The distribution coefficient (K_C_ = 1.16) of the target compound was determined according to [10] as the concentration of **1** in the lower stationary phase divided by that of the upper mobile phase.

#### 3.3.2. Fractionation of the Alkaloid Fraction (ALOF) by Centrifugal Partition Chromatography

The separation of the alkaloid fraction (4 g, sample concentration: 250 mg/9 mL) was carried out on a CPC-250 (Gilson, Limburg, Germany) chromatography system. The eluent system Hexane: EtOAc/MeOH:H_2_O (7:3/7:3) (*v*/*v*/*v*/*v*) was equilibrated in a separating funnel before application. In ascending mode (1200 rpm, 3 mL/min) portions of 6 mL were collected into test tubes, monitored by TLC and visualized with Dragendorff’s spray reagents (bismuth subnitrate (0.85 g):H_2_O (40 mL):acetic acid (10 mL):potassium iodide solution (400 g·L^−1^; 20 mL), European Pharmacopoeia Reagent). After termination of the elution mode (720 mL) the extrusion phase (400 mL) was also separated and recovered in test tubes by stopping the rotation and increasing the flow rate (5 mL/min) at the same time. The elution phase yielded 15 fractions and *O*-tigloylyclovirobuxeine-B was detected in the elution volume range from 54 to 96 mL (fraction 2; K_C_ range 0.1–0.29; note that the lower K_C_ value in comparison with the selection test above is likely due to the much higher concentration of **1** under these conditions, which may lead, e.g., to dimerization of the analyte [10]) by comparison with an authentic reference sample [5]. The content of **1** in fraction 2 was 11.7% (39.6 mg **1** in 337.7 mg of fraction 2 obtained from 4 g ALOF). The recovery rate was thus 86.8% and the enrichment factor of this separation step was 10.

#### 3.3.3. Subfractionation of Fraction 2 by Centrifugal Partition Chromatography

The following modifications were made to the fractionation of fraction 2 in contrast to 3.3.2. The biphasic solvent system Hexane/CH_3_CN:CH_2_Cl_2_ (10/7:3) (*v*/*v*/*v*) was used. This separation system had been used during our previous purification of **1** [13]. In the present work, the distribution coefficient (K_C_ = 0.63) in this solvent system was determined by LC/MS as above (2.4 mg of fraction 2 equilibrated in 2 mL of each of the two phases; see Figure S2, Supplementary Materials) and found suitable. The flow rate for the elution mode was set to 2.5 mL/min and rotation to 1300 rpm. The elution phase yielded 130 eluates (6.25 mL each). 310 mg of fraction 2 were separated in this way.

*O*-tigloylcyclovirobuxeine-B (**1**) was present almost quantitatively in pure form in the elution volume range from 93.75 to 106.25 mL (test tubes 16 and 17; K_C_ range 0.28–0.33; again, a much higher analyte concentration than in the previous experiment was the likely cause for the lower K_C_ values). Following crystallization from acetonitrile, 35 mg fine white needles of *O*-tigloylcyclovirobuxeine-B (**1**) were obtained. The purity was 91.1% as estimated from the LC/MS analysis (compare Figure 3). The recovery rate of this last purification step was 96.2% and the enrichment factor 7.8.

The overall enrichment of the three purification steps was thus 1482-fold with a total recovery rate of 76%.

The target compound (tR 5.39 min, [M + H]^+^: *m/z* 497.4116, [M + 2H]^2+^: *m/z* 249.2087) was accompanied by small amounts of two congeneric alkaloids with [M + H]^+^ at *m/z* 415 and 485 constituting about 3.9 and 4.9% (tR 3.77 and 5.13 min, Figure 3), as estimated by integration of extracted ion chromatograms for the respective [M + 2H]^2+^ ions. The former of these impurities was unambiguously identified as unesterified cyclovirobuxeine-B by its exact mass ([M + H]^+^: *m/z* 415.3731, calcd for C_27_H_47_N_2_O^+^ 415.3738; [M + 2H]^2+^: *m/z* 208.1923) and by comparison to authentic reference compound [13,14] The formation of this compound in small quantities by hydrolysis of **1** during the workup and isolation process cannot be excluded. The latter displayed an [M + H]^+^ at *m/z* 485.4179 corresponding to C_31_H_53_N_2_O_2_^+^ ([M + 2H]^2+^ at 243.2134). From the occurrence of the very intense doubly protonated ion; however, it can be expected to be a diaminocycloartanoid related to **1**, which cannot be characterized in more detail at present.

### 3.4. Spectroscopic Analysis of O-tigloylcyclovirobuxeine-B (1)

#### 3.4.1. NMR Spectroscopy

NMR spectra were recorded on Agilent DD2 600 MHz spectrometer (Agilent, Santa Clara, CA, USA) at 25 °C in CDCl_3_. Spectra were referenced to the solvent signals (^1^H: 7.260 ppm; ^13^C: 77.160 ppm) and were evaluated with MestReNova version 11.0 software (Mestrelab Research, Santiago de Compostela, Spain).

#### 3.4.2. UHPLC/+ESI-QqTOF-mass Spectrometry

UHPLC/+ESI-QqTOF-MS and MS/MS measurements were performed as described previously [5] with minor modifications. The solvent flow rate was set at 0.4 mL/min. Water (with 0.1% formic acid; A) and acetonitrile (with 0.1% formic acid; B) were used as mobile phase. The binary gradient adopted was as follows: 0–1.88 min: linear from 15% B to 30% B; 1.88–7.88 min: linear from 30% B to 33% B; 7.88–9.9 min: linear from 33% B to 50% B; 9.9–9.93 min: linear from 50% B to 100% B; 9.93–15.88: isocratic 100% B; 15.88–15.98 min: linear from 100% B to 15% B; 15.98–20.0 min: isocratic 15% B. The injection volume of pure compound was 1 µL.

Sample concentrations: GBUS (10 mg/mL), ALOF (10 mg/mL), fraction 2 (1 mg/mL), *O*-tigloylcyclovirobuxeine-B (**1**) (0.1 mg/mL).

Quantification of the target compound was carried out based on peak integration in extracted ion chromatograms of the intense [M + 2H]^2+^ ion. A calibration line (see Figure S9, Supplementary Materials, R^2^ = 0.9958) was obtained from six different concentrations of the purified compound (10, 8, 6, 4, 2 and 1 µg/mL), each analysed three times. Samples of GBUS, ALOF, fraction 2 and the samples for determination of K values during selection of CPC solvent systems were diluted to appropriate concentration within the range of the calibration line. All calculated concentrations were corrected by the purity factor of 0.91.

To study the fragmentation of **1**, a separate experiment in MRM mode was carried out with the purified compound, pre-selecting the [M + H]^+^ ion at *m/z* 497.42 ± 10 and a collision energy of 40 eV.

### 3.5. Spectral Data of O-tigloylcyclovirobuxeine-B (1)

*O*-tigloylcyclovirobuxeine-B (**1**): white crystals; ^1^H NMR (600 MHz, CDCl_3_; δ (ppm), intensity, mult., J (Hz)): 6.84 (1H, qq, 7.1, 1.5, H-3`), 5.60 (1H, ddd, 10.7, 1.4, 1.3, H-6), 5.37 (1H, ddd, 10.3, 6.1, 3.1, H-7), 5.10 (1H, ddd, 8.5, 6.0, 1.1, H-16), 2.67 (1H, m, H-20), 2.57 (1H, dd, 6.2, 2.5, H-8), 2.35 (3H, s, H-33), 2.30 (6H, s, H-31/32), 2.14 (1H, m, H-15), 2.12 (1H, m, H-17), 2.04 (1H, m, H-3), 1.84 (1H, m, H-11), 1.83 (1H, m, H-5), 1.81 (3H, dq, 1.4, H-5`), 1.76 (3H, dq, 7.1, 1.3, H-4`), 1.76 (1H, m, H-2), 1.74 (1H, m, H-12), 1.54 (2H, m, H-1), 1.54 (1H, m, H-2), 1.49 (1H, m, H-12), 1.44 (1H, m, H-11), 1.24 (1H, d(d), 14.2, (<1), H-15), 1.10 (3H, d, 6.2, H-21), 1.04 (3H, s, H-29), 1.00 (3H, s, H-18), 0.94 (3H, s, H-28), 0.79 (3H, s, H-30), 0.72 (1H, d, 4.1, H-19), −0.18 (1H, d, 4.1, H-19);

^13^C NMR (150 MHz, CDCl_3_; δ (ppm)): 168.08 (qC, C-1`), 137.82 (qC, C-2`), 128.87 (CH, C-3`), 128.17 (CH, C-7), 127.98 (CH, C-6), 80.41 (CH, C-16), 71.55 (CH, C-3), 57.37 (CH, C-20), 57.03 (CH, C-17), 49.52 (qC, C-14), 48.85 (CH, C-5), 46.18 (qC, C-13), 44.31 (CH_3_, C-31/32), 43.13 (CH, C-8), 42.77 (CH_2_, C-15), 41.55 (qC, C-4), 33.24 (CH_3_, C-33), 32.28 (CH_2_, C-12), 31.13 (CH_2_, C-1), 28.73 (qC, C-10), 26.18 (CH_3_, C-29), 25.11 (CH_2_, C-11), 20.73 (qC, C-9), 20.06 (CH_2_, C-2), 18.51 (CH_3_, C-21), 18.18 (CH_2_, C-19), 17.57 (CH_3_, C-28), 16.58 (CH_3_, C-30), 15.98 (CH_3_, C-18), 14.56 (CH_3_, C-4`), 12.17 (CH_3_, C-5`).

+ESI-QqTOF-MS (*m/z*): 497.4116 [M + H]^+^, 249.2134 [M + 2H]^2+^ (calcd for C_32_H_53_N_2_O_2_^+^: 497.4102, for C_32_H_54_N_2_O_2_^2+^: 249.2087). MS/MS data for [M + H]^+^ are reported in Figure 5.

The spectral data were in full agreement with findings reported in literature [5].

## 4. Conclusions

The present work reveals a state-of-the-art semi-preparative scale isolation method of *O*-tigloylcyclovirobuxeine-B (**1**), a compound with promising antiplasmodial activity. In comparison to previously described extractions of this natural compound [5,15] we have not used any solid packing material. Thereby we achieved a high recovery and purification of our sample during isolation. Our findings demonstrate that CPC is an advantageous chromatographic technique for natural compound isolation also in case of amino *nor*-cycloartane-type alkaloids from *Buxus*. The target compound of our isolation, *O*-tigloylcyclovirobuxeine-B (**1**), has the potential to serve as a lead compound against *Plasmodium falciparum*. Since *B. sempervirens* is a very popular ornamental plant that requires periodic trimming, its leaves represent a very sustainable source of this valuable compound. Studies on temporal variability of the content of **1** in *B*. *sempervirens* leaves are in progress, in order to identify an optimal harvesting time to further increase the yield. Consequently, the isolation method presented here is an important step in obtaining sufficient quantities of this interesting alkaloid for further in-depth studies with the aim of designing analogues with improved activity and toxicity profiles.

## Figures and Tables

**Figure 4:**
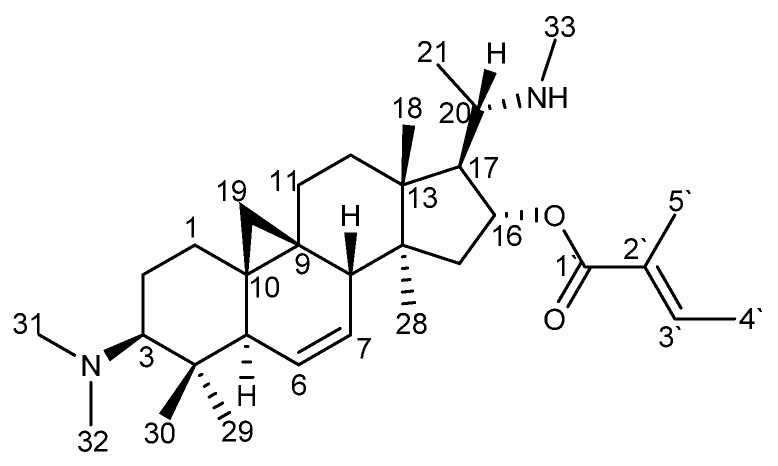
Chemical structure of *O*-tigloylcyclovirobuxeine-B (**1**).

## Supplementary Materials

The following are available online, Table S1: Solvent system selection process for the alkaloid fraction (ALOF). Figure S1: Determination of a suitable phase system for the fractionation by TLC. Figure S2: Determination of a suitable phase system for the fractionation of fraction 2 by UHPLC/+ESI-QqTOF-MS. Figure S3: ^1^H NMR spectrum of *O*-tigloylcyclovirobuxeine-B (**1**) (600 MHz, CDCl_3_). Figure S4: Detail of the ^1^H NMR spectrum of *O*-tigloylcyclovirobuxeine-B (**1**) (600 MHz, CDCl_3_). Figure S5: ^13^C NMR spectrum of *O*-tigloylcyclovirobuxeine-B (**1**) (150 MHz, CDCl_3_). Figure S6: Detail of the ^13^C NMR spectrum of *O*-tigloylcyclovirobuxeine-B (**1**) (150 MHz, CDCl_3_). Figure S7: +ESI/QqTOF mass spectrum (full scan) of *O*-tigloylcyclovirobuxeine-B (**1**). Figure S8: +ESI/QqTOF MS/MS spectrum of the [M + H]^+^ ion of *O*-tigloylcyclovirobuxeine-B (**1**). Figure S9: Calibration line for the quantitive determination of *O*-tigloylcyclovirobuxeine-B (**1**).
