# Supplementary material for: Target-Guided Isolation of O-tigloylcyclovirobuxeine-B from Buxus sempervirens L. by Centrifugal Partition Chromatography
Source: Molecules. 2020 Oct 19;25(20):4804. doi: 10.3390/molecules25204804 (PMC7587941; doi:10.3390/molecules25204804)
Supplement: Supplementary file 1 [file molecules-25-04804-s001.pdf]

molecules

Supplementary Material

Target-Guided Isolation of *O*-tigloylcyclovirobuxeine -B from *Buxus sempervirens* L. by Centrifugal Partition Chromatography

Lara U. Szabó [1] and Thomas J. Schmidt [1,*]

[1] Institute of Pharmaceutical Biology and Phytochemistry (IPBP), University of Münster, Pharma Campus Correnstraße 48, D-48149 Münster, Germany; lszabo@uni-muenster.de
* Correspondence: thomschm@uni-muenster.de; Tel.: +49-251-83-33378

Table S1. Solvent system selection process for the alkaloid fraction (ALOF).

Solvent system	Observation
Chloroform : MeOH : PrOH : H_2O : NH_4OH (35 : 65 : 40 : 5 : 1)	No phase separation
Chloroform : MeOH : PrOH : H_2O : NH_4OH (35 : 65 : 5 : 40 : 1)	Emulsification
Hexane / ACN : DCM (10 : 7 : 3)	Enrichment in lower phase (TLC)
Hexane / EtOH : H_2O (6 : 5 : 1)	Enrichment in lower phase (TLC)
Hexane : EtOAc / MeOH : H_2O (7 : 3 : 7 : 3)	Equal distribution between the two phases (TLC) → K value determination by LC/MS

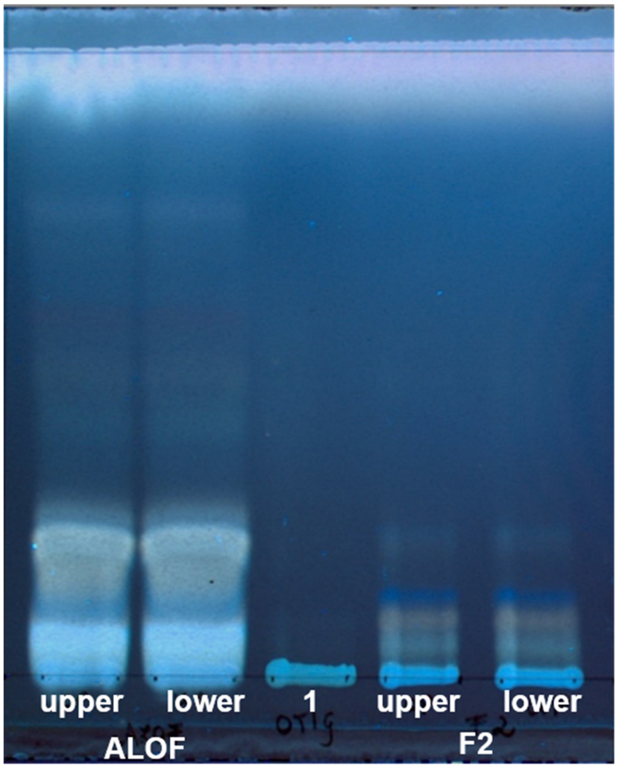

Figure S1. Determination of a suitable phase system for the fractionation by TLC. TLC plate of ALOF, O-tigloylcyclovirobuxeine-B (**1**) and fraction 2 (F2) visualized with Liebermann–Burchard spray reagents (366 nm). Note that these TLC separations were performed in order to check for equal distribution of ALOF and fraction 2 between the two phase, not for an optimal separation of these samples.

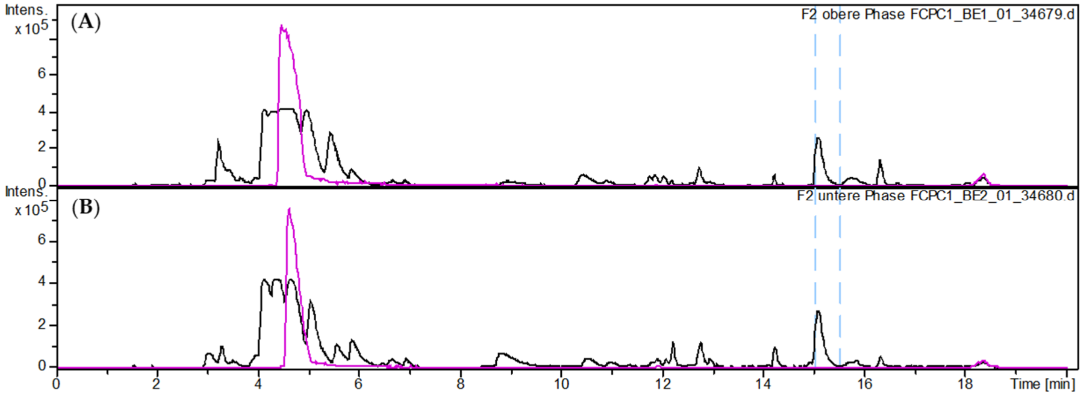

Figure S2. Determination of a suitable phase system for the fractionation of fraction 2 by UHPLC/+ESI-QqTOF-MS. Base peak chromatogram of *m/z* 200-1000 (black), Extracted ion chromatogram of *m/z* 497 $[M + H]^+$ (pink); (**A**) upper phase; (**B**) lower phase.

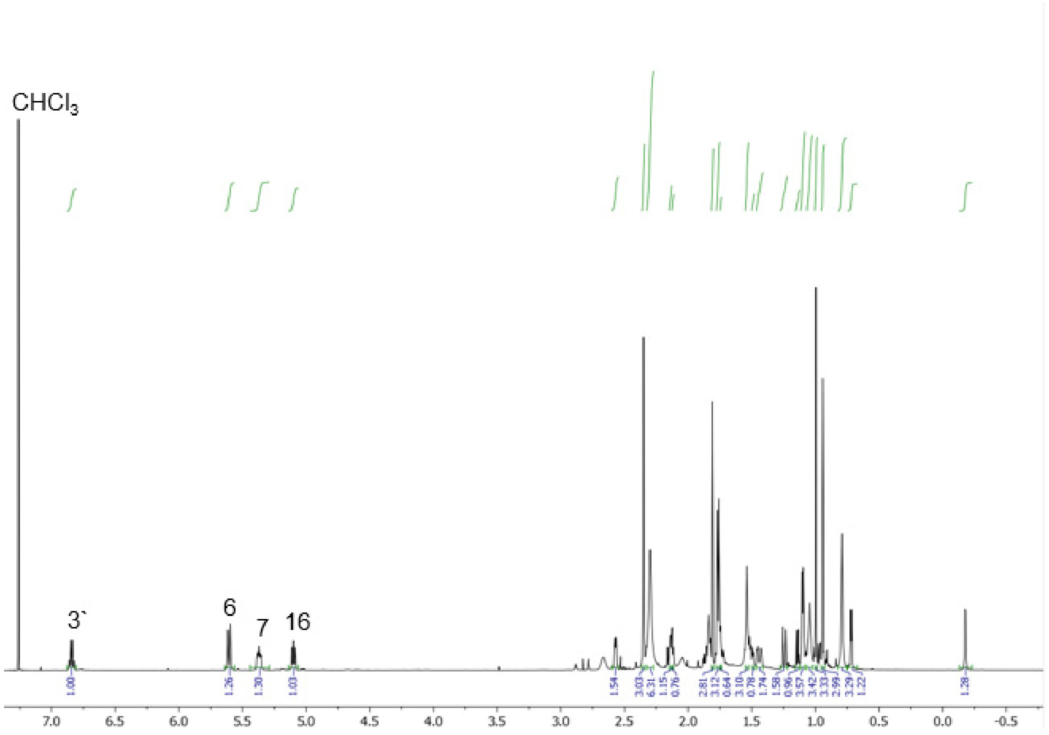

Figure S3. ^{1}H NMR spectrum of O-tigloylcyclovirobuxeine-B (**1**) ($CDCl_3$, 600 MHz). The assignment of the signals between -0.5 and 3 ppm can be found in the expanded region shown in Figure S4.

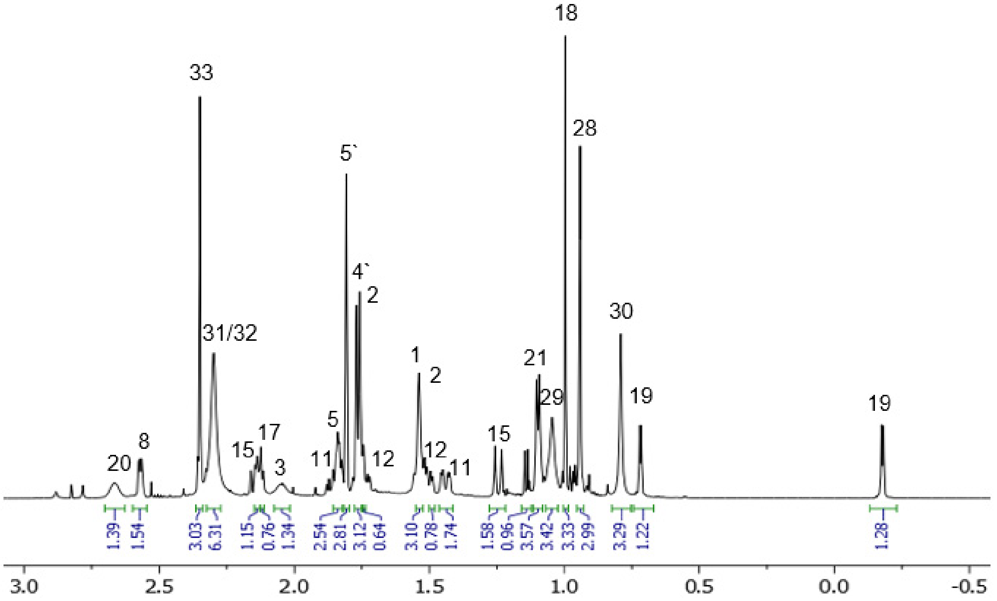

Figure S4. Detail of the ^{1}H NMR spectrum of O-tigloylcyclovirobuxeine-B (**1**) ($CDCl_3$, 600 MHz).

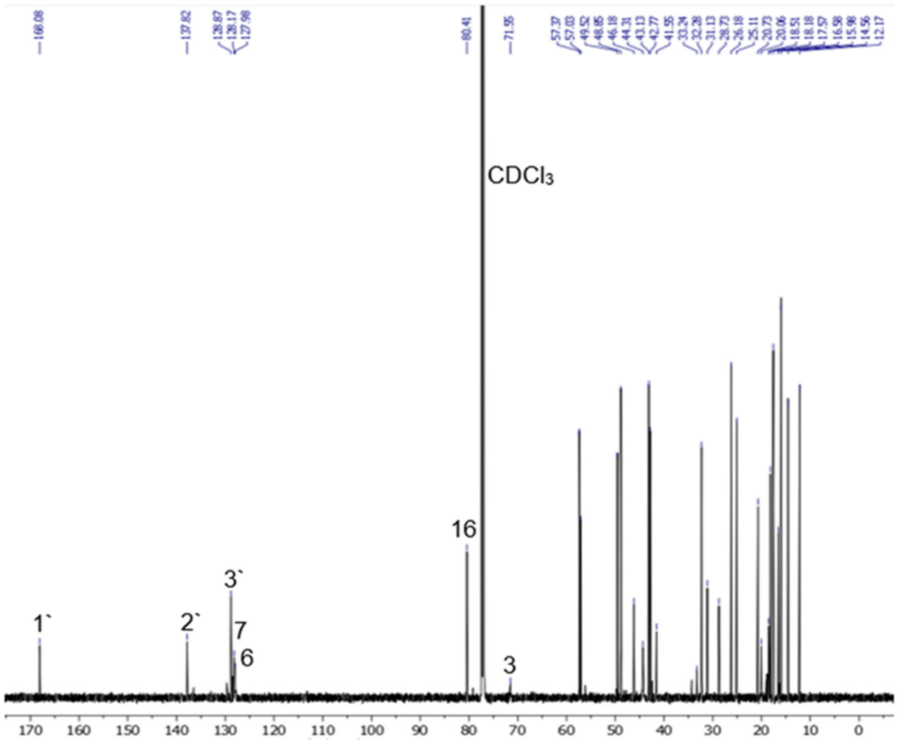

Figure S5. ^{13}C NMR spectrum of O-tigloylcyclovirobuxeine-B (**1**) ($CDCl_3$, 150 MHz). The assignment of the signals between 10 and 60 ppm can be found in the expanded region shown in Figure S6.

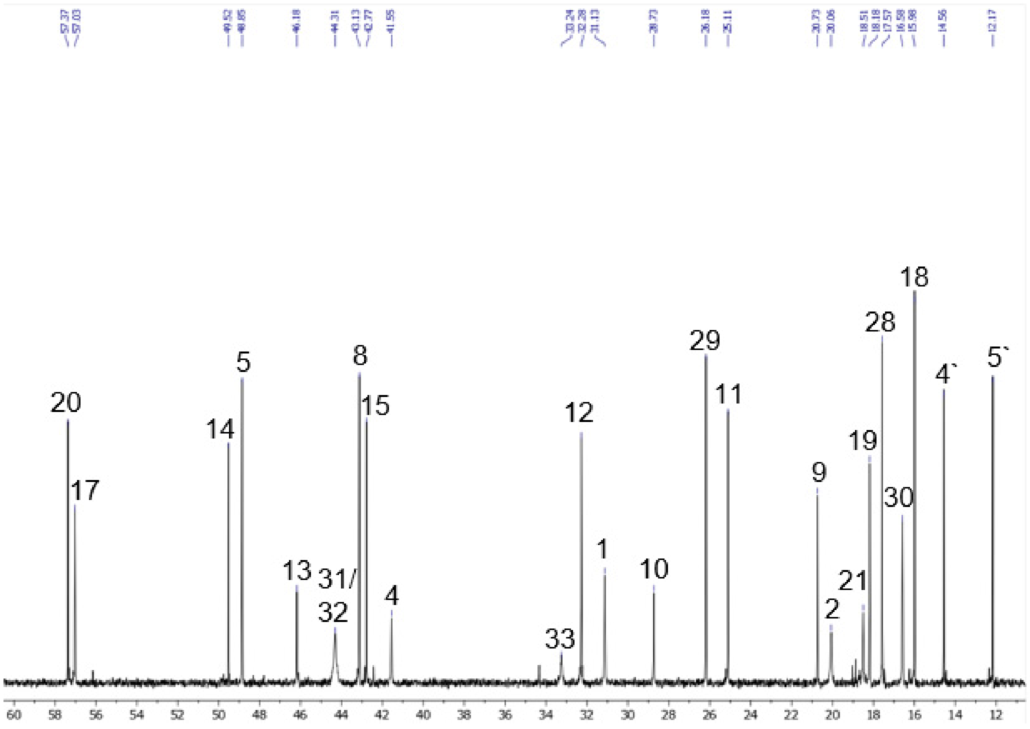

Figure S6. Detail of the ^{13}C NMR spectrum of O-tigloylcyclovirobuxeine-B (**1**) ($CDCl_3$, 150 MHz).

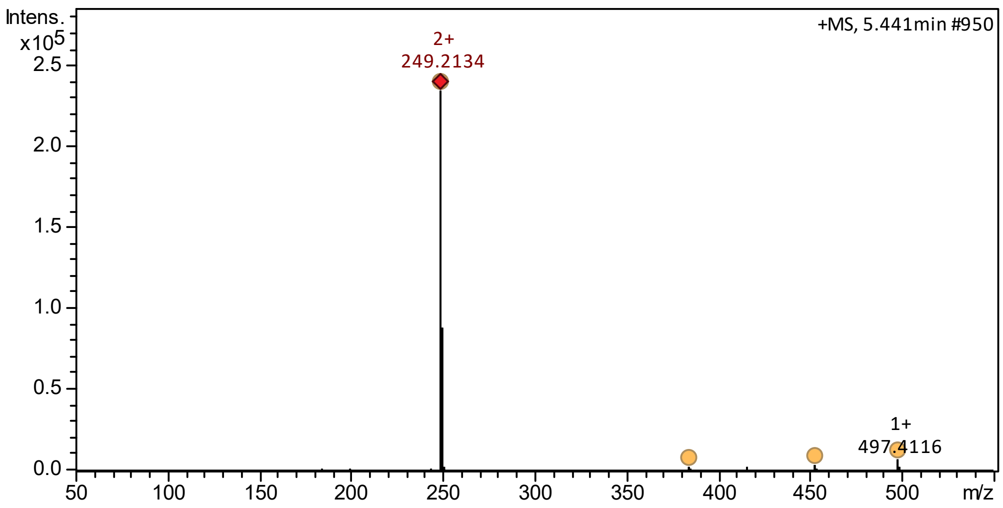

Figure S7. +ESI/QqTOF mass spectrum (full scan) of O-tigloylcyclovirobuxeine-B (**1**).

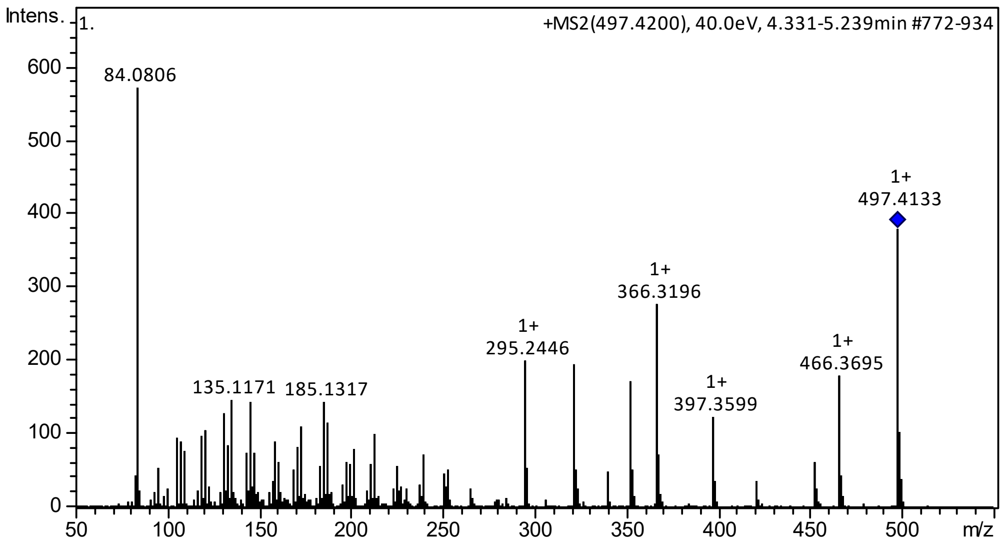

Figure S8. +ESI/QqTOF MS/MS spectrum of the $[M + H]^+$ ion of O-tigloylcyclovirobuxeine-B (**1**). An expanded region of this spectrum is shown in Figure 5A of the main manuscript.

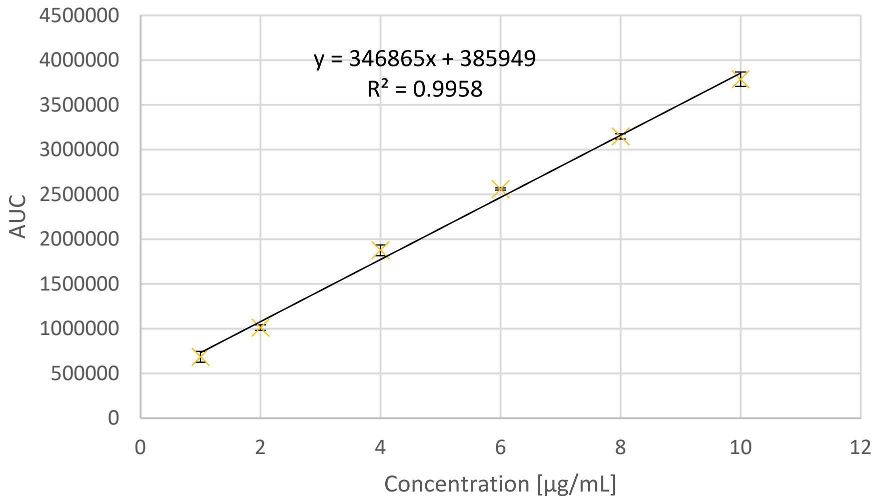

Figure S9. Calibration line for the quantitively determination of O-tigloylcyclovirobuxeine-B (**1**). Data were obtained by integration of LC/MS chromatograms (extracted ion chromatograms of the most stable ion, $[M+2H]^{2+}$). Each point represents the mean of three analyses with error bars showing the standard deviation.
